# Fenretinide-induced Apoptosis of Acute Myeloid Leukemia Cells via NR4A1 Translocation into Mitochondria and Bcl-2 Transformation

**DOI:** 10.7150/jca.32167

**Published:** 2019-11-01

**Authors:** Jie Xiong, Xingyi Kuang, Tingting Lu, Xu Liu, Bingqing Cheng, Weili Wang, Danna Wei, Xinyao Li, Zhaoyuan Zhang, Qin Fang, Depei Wu, Jishi Wang

**Affiliations:** 1 Jiangsu Institute of Hematology, The First Affiliated Hospital of Soochow University, Key Laboratory of Thrombosis and Hemostasis under Ministry of Health, Collaborative Innovation Center of Hematology, Suzhou Institute of Blood and Marrow Transplantation,188 Shizi Street, Suzhou 215006, Jiangsu, China; 2 Department of Hematology, The Affiliated Hospital of Guizhou Medical University. Hematopoietic Stem Cell Transplantation Center of Guizhou Province, Key Laboratory of Hematological Disease Diagnostic & Treat Centre of Guizhou Province. Guizhou Medical University, Guiyang 550001, China.; 3 Department of Critical Care Medicine, Affiliated Hospital of Guizhou Medical University, Guiyang 550001, China; 4 Department of Pharmacy, Affiliated Hospital of Guizhou Medical University, Guiyang 550001, China.

**Keywords:** NR4A1, Fenretinide, Acute myeloid leukemia, Apoptosis, Nuclear export

## Abstract

**OBJECTIVE:** Fenretinide is reported to induce NR4A1-associated apoptosis in several types of cancer cells. However, it remains unclear about its specific role and the underlying mechanism in acute myeloid leukemia (AML). Therefore, this study aimed to explore the role and mechanism of fenretinide-induced apoptosis in AML.

**METHOD**: Firstly, the NR4A1 mRNA level in the newly diagnosed AML patients was measured, then AML cells were treated with fenretinide at various time points and doses, and cell viability was investigated by using the cell-counting kit-8 (CCK-8) assay. Additionally, apoptosis and cell cycles were analyzed by using flow cytometry. Moreover, siNR4A1 was utilized to knockdown NR4A1 expression, and leptomycin B (LMB) was adopted to inhibit the nuclear export; afterwards, the apoptosis rate and expression of apoptotic proteins in AML cells were detected. In addition, the expression levels of NR4A1 in the nuclei and mitochondria of fenretinide-treated AML cells were also measured. Meanwhile, the interaction between NR4A1 and Bcl-2, as well as the Bcl-2 transformation, was also examined. The anti-leukemic effect of fenretinide on NOD/SCID mice was also determined through subcutaneous injection of HL-60 cells.

**RESULTS:** NR4A1 expression in AML patients was markedly down-regulated compared with that in normal donors. Fenretinide induced the expression of NR4A1 and mitochondria-mediated apoptotic pathway-associated proteins in a time- and concentration-dependent manner. Importantly, both siNR4A1 alone or the combination of fenretinide with LMB could attenuate the fenretinide-induced apoptosis and expression of apoptotic proteins. Under the action of fenretinide, the NR4A1 protein expression was down-regulated in nuclear extracts whereas up-regulated in mitochondrial extracts. At the same time, fenretinide promoted NR4A1 translocation from nuclei into mitochondria, and enhanced the interaction between NR4A1 and Bcl-2, thereby exposing the BH3 domain of Bcl-2 to exert the anti-apoptotic effect. Moreover, fenretinide also exhibited an anti-leukemic effect and induced NR4A1 expression in the AML mouse model.

**CONCLUSIONS:** Fenretinide exerts an obvious effect on AML cells both *in vitro* and *in vivo.* Besides, the NR4A1-mediated signaling pathway is highly involved in the fenretinide-induced apoptosis of AML cells.

## Introduction

Acute myeloid leukemia (AML) represents a group of heterogenous hematopoietic malignancy with frequent relapses and poor outcomes^1^ . The US has witnessed almost 20,000 newly diagnosed AML cases in 2018^2^. Typically, the median age at diagnosis is 67 years old, and 30% patients are diagnosed at an age of > 75 years 3. Additionally, about 70% older patients die within 1 year of diagnosis, and long-term survival is rare, which is experienced only by about 5% patients^4^-^7^. Although improvements have been made in prognostic stratification and diagnosis of AML, treatment to achieve and maintain first complete remission (CR1) has remained nearly unchanged for the last four decades^8^. Allogeneic hematopoietic stem cell transplantation (allo-HSCT) is a potential treatment for AML. Nevertheless, the advanced patient age and donor sources have limited the use of allo-HSCT in AML. As estimated, the number of AML patients is to rise as a result of the increased population aging. Therefore, it is very important to search for the new alternative treatments for AML.

NR4A1 (also referred to as Nur77) belongs to the highly-conserved nuclear orphan receptor family of proteins, which are activated by a large number of stimuli^9^,^10^. NR4A1 is also implicated in regulating the proliferation, apoptosis and cell cycle arrest of cancer cells^11^. Plenty of evidence suggests that NR4A1 is over-expressed in numerous cancer types, including pancreatic, bladder, colon, and cervical cancers as well as melanoma, where it exhibits the pro-oncogenetic activity and enhances cell survival and/or proliferation^12^. Furthermore, the deletion of NR4A1 and NR4A3 in mice is found to lead to AML^13^, and redujinction in the gene dosages of NR4A1 and NR4A3 in hypoallelic mice beyond a critical threshold is sufficient to cause mixed myelodysplastic/myeloproliferative neoplasms (MDS/MPN), along with AML progression^14^. Together, these findings suggest that targeting NR4A1 may be an appropriate alternative therapy for myeloid malignancies.

Fenretinide [also named as N-(4-hydroxyphenyl) retinamide, 4-HPR] is a synthetic derivative of all-trans retinoic acid (ATRA). It has been verified in numerous experimental and clinical studies to play a potential role in chemoprevention and therapy^15^. Typically, it can induce the apoptosis of various cancer cell types *in vivo*, including liver, lung, bladder, and prostate cancers, together with lymphoma^15^-^19^, thereby impairing cancer cell migration and invasion^20^; additionally, it is also used *in vitro*, both in animal models and in the clinical setting. Moreover, fenretinide has also been utilized in clinical trials of breast, bladder, ovarian, recurrent small cell lung cancers, as well as neuroblastoma, cervical neoplasia, relapsed/refractory peripheral T-cell lymphoma, and B-cell lymphoma^16^,^21^-^27^. Nevertheless, the effect of fenretinide on AML cells, together with its underlying mechanism, has rarely been reported. It is shown that fenretinide can induce NR4A1 expression in Huh-7 hepatoma cell line^15^. Nonetheless, it remains unknown about whether fenretinide induce the apoptosis of AML cell line via NR4A1.

The present study was therefore undertaken to test the effect of fenretinide on the proliferation and apoptosis of AML cell lines, via inducing NR4A1 and its relocalization from the nucleus into mitochondria, along with the subsequent Bcl-2 transformation.

## Materials and Methods

### Patient characteristics

Bone marrow was obtained from newly-diagnosed AML patients at the Hematopoietic Stem Cell Laboratory of the Affiliated Hospital of Guizhou Medical University from October 2017 to January 2018. All patients signed written informed consents according to the Declaration of Helsinki and the study was approved by the institutional review board (Affiliated Hospital of Guizhou Medical University). The characteristics of the patients and donors are summarized in Table 1. Bone marrow mononuclear cells (MNCs) were isolated by Ficoll density centrifugation to extract mRNA or protein.

### Reagents

All reagents and chemicals used were kindly offered by Hematopoietic Stem Cell Transplantation Center Laboratory of Guizhou Medical University unless noted otherwise. Fenretinide was purchased from MedChem Express, dissolved in DMSO and stored at -80℃. Leptomycin B was purchased from Solarbio Science&Technology Co.(Beijing,China). The antibodies against cleaved caspase 3, cleaved caspase 9, cleaved PARP and Bax were obtained from Santa Cruz Biotechnology (CA, USA). The antibodies against β-actin, mouse or rabbit IgG were purchased from Beyotime Biotechnology Co. (Shanghai, China). The antibodies against NR4A1 and Cytochrome C (cyt-c) were purchased from MDL biotech Co.(Beijing, China).

### Cell Culture

AML cell lines HL-60 and Kasumi-1 cells were maintained in RPMI-1640 medium (Gibco Life Technologies, Carlsbad, CA, USA) with 10% fetal bovine serum (Invitrogen, USA) and 1% penicillin/streptomycin (Invitrogen, USA). All cells were maintained in a 37°C incubator with 95% humidity and 5% CO_2_. All experiments were conducted using logarithmically growing cells (3-6 × 10^5^ cells/ml).

### Cell viability Assay

The inhibitory effects were assessed by using the cell counting kit assay (CCK-8 assay). Cells were seeded at a density of 4,000 cells/well in 96-well plates with or without fenretinide treatment in culture medium, the cells were treated for 12, 24 and 48 hours (h) , respectively, 20 μl of the CCK8 solution was then added to each well. After 2 h of incubation at 37°C, the absorbance at 450 nm was measured using a spectrophotometer (Molecular Devices, Sunnyvale, California, USA).

### Analysis of Apoptosis

Apoptosis was determined by annexin V-FITC and propidium iodide double staining. Cells were seeded at a concentration of 2 ×10^5^ cells/ml and incubated for 48 h with fenretinide at various concentrations. After treatment, cells were harvested, washed with PBS and stained with the annexin-V/propidium iodide apoptosis kit (7Sea Biotech, Shanghai, China) according to manufacturer's instructions. Flow cytometry analyses were performed on a BD LSRFortessa Cell Analyzer (BD Biosciences, San Jose, CA, USA). Data were analyzed using Flow Jo 7.6.1 software (Tree Star, Inc., Ashland, OR, USA).

### RNA isolation and reverse transcription PCR

RNAs were extracted from cell lines and primary MNCs samples using Trizol reagent (Invitrogen) according to the manufacturer's instructions. qRT-PCR was performed using an SYBR Green PCR Master Mix (TianGen Biotech, Beijing, China) and a PRISM 7500 Real-Time PCR System (ABI, ABI PRISM, USA). Gene expression levels were analyzed relative to the level of the β-actin gene transcript. The primers for qPCR were as follows: NR4A1, F: 5′-CGGCTACCTTCAAAACCCAA-3′, NR4A1, R: 5′-CATAAAATTGTTGCACGT CACC-3′, β-actin, F: 5′-CTACCTCATGAAGATCCTCACCGA-3′; β-actin, R: 5′-TTCTCCTTAATGTCACGCACGATT-3′. cDNA samples were mixed with the primers and SYBR Master Mix in a total volume of 20 μl. The thermal cycling conditions used in the protocol were 10 min at 95°C, followed by 40 cycles at 95°C for 10s, 60°C for 30 s and 72°C for 32s.

### Western blotting analysis and Grey value analysis

Cells from different groups were harvested, washed in PBS and lysed in RIPA buffer with 1 μM PMSF (Solarbio cience & Technology, Beijing, China), then stilled at 4°C for 30 min followed by centrifugation for 10 min. Supernatants were loaded on 10% SDS-PAGE gel and the separated proteins transferred onto polyvinylidene fluoride membrane (Millipore Corporation, Milford, Massachusetts, USA), which was then blocked in 10% nonfat milk in Tris buffer for 2 h with agitation and washed. Then, the membrane was blotted with primary antibodies for 2 h. After washing, the membrane was incubated with secondary antibodies (HRP-conjugated goat anti-rabbit or anti-mouse; Beyotime) for 45 min at room temperature. All protein bands were visualized with the use of the enhanced chemiluminescence (7Sea Biotech). According to manufacturer's instructions, cytoplasmic proteins were extracted using the Beyotime cytoplasmic protein extraction kit, and nucleoproteins were extracted using the Beyotime nucleoproteins protein extraction kit. Equal amounts of protein lysate were used for western blotting analysis. All tests were repeated three times. Grey value analysis was determined by Quantity One 4.6.2.

### Silencing by siRNA transfection

NR4A1 siRNA (China Quan Yang Biological Co., Ltd., Shanghai) was diluted into different concentrations, and transduction was conducted using electrotransducer (Invitrogen, USA) according to the manufacturer's instructions. The human siNR4A1 target is 5′-TACACAGGAGAGTTTGACA-3′. Real-time PCR and western blot was used to detect the silencing of NR4A1 expression.

### Immunoprecipitation (IP) Assay

The interaction between NR4A1 and Bcl-2 proteins in HL-60 cells at 12 h after fenretinide treatment was tested by IP according to the manufacturer's instructions (Beyotime, Shanghai, China). HL-60 cells (4 × 10^6^) were washed with PBS and lysed with RIPA lysis buffer for 20 min on ice. Then, the lysate was collected after centrifugation at 12,000 rpm for 10 min at 4 °C. Magnetic beads were incubated with Anti-Bcl-2 and anti-rabbit IgG antibodies at 4 °C for 3 h. After three washes with PBS, according to standard protocols, bead-bound proteins were analyzed by western blotting.

### Immunofluorescence Staining

After treatment, HL-60 cells were harvested and centrifuged, then fixed with 4% paraformaldehyde in PBS for 30 min and washed with PBS 3 times. Cell membrane permeabilization was performed with 0.1% Triton-X100 for 10 min. Afterwards, cells were incubated with fresh goat serum (5%) in following 2 h, cells were probed with specific primary antibodies against Bcl-2 BH3 at 4 °C overnight. After washing with PBS 3 times again, cells were incubated with the corresponding fluorescent-labeled secondary antibodies. Finally, DAPI was used to stain the nuclei. Fluorescence images were captured under a fluorescence microscope (Leica DM4000B, Wetzlar, Germany).

### Mouse xenografts

Twelve 4 week - old NOD/SCID mice (six male and six female) were purchased from the Institute of Laboratory Animal Sciences (PUMC, Beijing, China). The mice were housed under specific pathogen-free conditions and allowed to acclimate for 2 weeks before experiments. The mice were housed (4 individuals per cage) and used at a weight of approximately 17-20 g. They were injected subcutaneously with 10 million cells of HL-60 (cell line). Tumor size and mouse weight were measured three times weekly; tumor volumes were calculated as 0.5×height×width×length^28^. Mice with progressively growing tumors between 100 and 300 mm^3^ were randomized into three groups. Fenretinide-LXS powder was administered by gavage (180 mg/kg/day, slurried in water, two divided doses daily for one week), ketoconazole(KETO) (38 mg/kg/day, dissolved in water, administered by gavage for five days per week). Controls were treated with the normal saline(NS). The study was terminated after 10 days of initiating treatment, and all surviving mice were sacrificed.

### TUNEL Assay

TUNEL assay was used for detection of apoptosis rate, a 4-micron paraffin section was prepared and apoptosis in the tumors of each group was determined by in situ Fluorescein cell death detection kit (Yisheng-40306ES60, Shanghai, China) according to the manufacturer's protocols. The number of apoptosis-positive cells was counted in five high-power fields (400× magnification), the mean percentage of apoptotic cells was showed.

### Statistical Analysis

All data graphs were drawn by GraphPad Primer 5. Data were represented as mean ± SEM. P<0.05 (two sided) was considered statistically significant. Statistical analysis was performed by the nonparametric tests for all continuous variables.

## Results

### Expression of NR4A1 in AML patients

**Figure 1:**
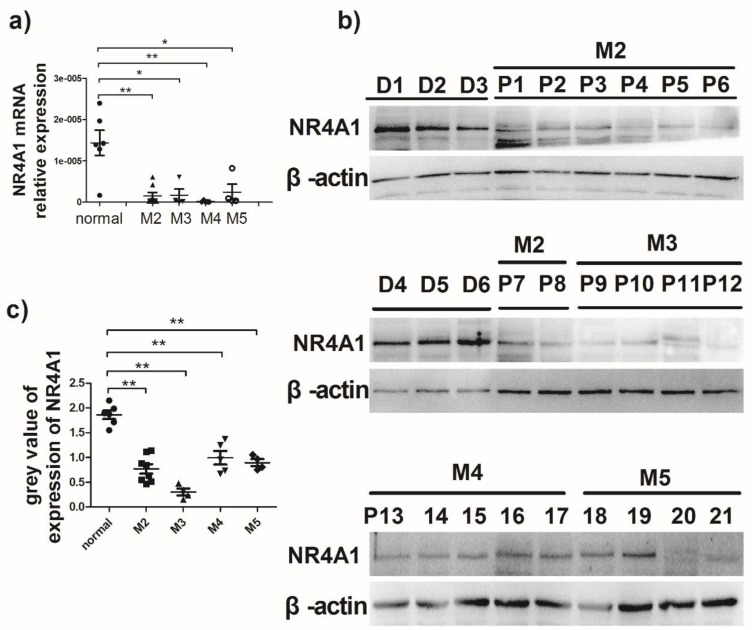
** Expression of NR4A1 in AML patients. a)** NR4A1 mRNA expression detected using real-time PCR. Data are expressed as mean ± SEM. The nonparametric test is used for statistical analysis. *p≤0.05; **p≤ 0.01; ***p≤0.001. **b)** The NR4A1 protein levels detected in donor group (n = 6) and patient group (n = 21). β-actin is used as the internal reference. c) Grey value of NR4A1 expression in patients.

It is previously reported that NR4A1 expression in AML patients is lower than that in healthy bone marrow donors^13^. To evaluate NR4A1 expression in AML patients, the newly diagnosed AML patients were recruited from the Hematopoietic Stem Cell Laboratory of the Affiliated Hospital of Guizhou Medical University from October 2017 to January 2018. The NR4A1 expression levels in AML patients were measured using qRT-PCR and Western blotting, respectively. Information on patients and healthy donors is presented in Table 1. Figure 1a shows that NR4A1 mRNA expression in the AML patients is markedly lower than that in healthy donors. Similarly, the NR4A1 protein expression in AML patients was found to be lower than that in healthy donors (Fig. 1b, 1c), regardless of their cytogenetic profile.

### Fenretinide inhibited the growth and induced the apoptosis of HL-60 and Kasumi-1 cells

**Figure 2:**
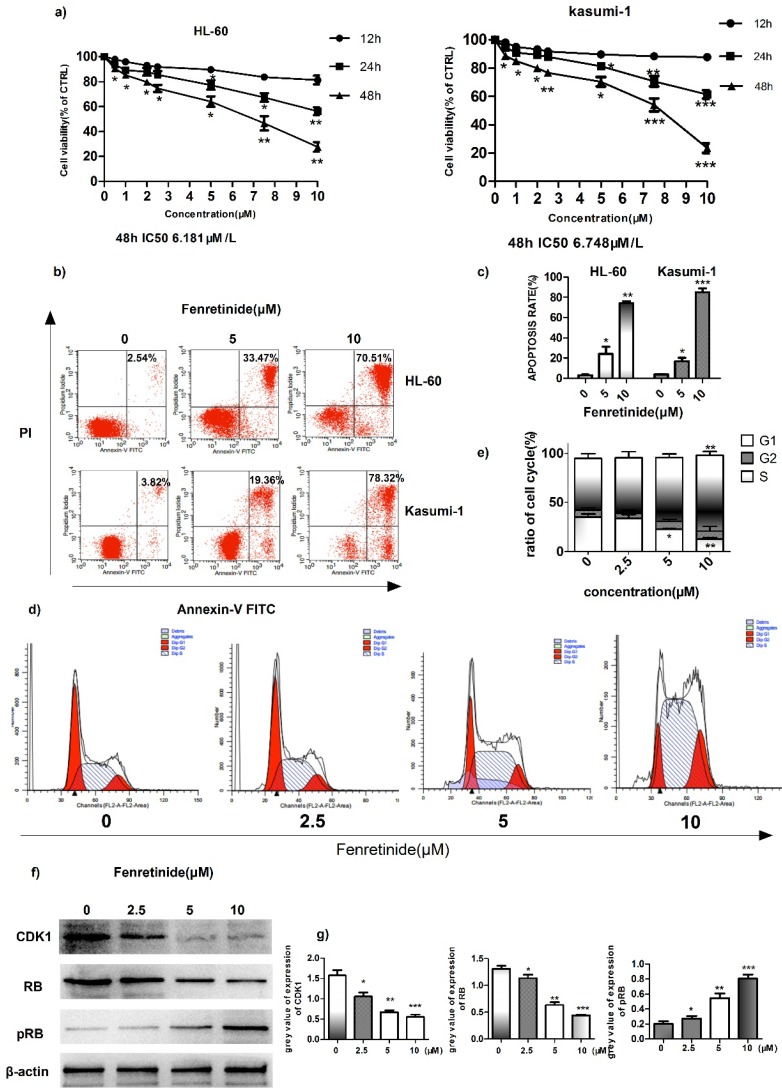
** Fenretinide inhibits cell growth and induces apoptosis of HL-60 and Kasumi-1 cells. a)** Cell viability. Cells are treated with fenretinide as indicated for 48 h. Cell viability is assessed according to the CCK-8 assay. **b-c)** Fenretinide induces the apoptosis of both HL-60 and Kasumi-1 cells. Cells are treated with fenretinide as indicated for 48 h. Apoptotic cells are tested using flow cytometry. **d-e)** HL-60 cells are treated with fenretinide at the concentrations of 0, 2.5, 5 and 10 μM for 12 h. The cell cycle is detected by flow cytometry. **f)** Cell lysates are collected for Western blotting, so as to analyze the expression of related cyclins. g) Grey values of cyclins expression in f), using Quantity One 4.6.2. All data represents the means from three independent experiments. *p≤0.05; **p≤ 0.01; ***p≤0.001 vs control.

To investigate the inhibition effect of fenretinide on cell growth, two AML cell lines, HL-60 and Kasumi-1, were treated with fenretinide (0.5 to 10 μM) for 12 to 48 h. Then, cell viability was evaluated according to the CCK-8 assay. As shown in Figure 2a, fenretinide treatment for 48 h evidently inhibits the viability of HL-60 and Kasumi-1 cells in a concentration-dependent manner (0.5 to 10 μM). After probing with Annexin V-FITC/PI, we determined the apoptosis rates of AML cell lines (HL-60 and Kasumi-1 cells) caused by fenretinide through flow cytometry (Fig. 2b, 2c). The apoptosis rates of both cell lines were increased after fenretinide treatment (10 μM) for 48 h. Notably, change in the cell cycle is one of the underlying mechanisms of cell apoptosis. To examine the stage at which HL-60 cells were blocked by fenretinide, flow cytometry analysis was conducted to determine the stage of cell cycle. Our results found that, 4-HPR markedly increased the percentage of HL-60 cells at S-phase in a dose-dependent manner (Fig. 2d, 2e). The expression levels of proteins involved in cell cycle regulation, including CDK1, RB and p-RB, were also measured. It was discovered that 4-HPR treatment down-regulated CDK1 and RB expression, whereas up-regulating p-RB expression in HL-60 cells (Fig. 2f, 2g), which was associated with cell cycle arrest at S-phase.

### Fenretinide-induced apoptosis was mediated by NR4A1 and a mitochondrial-dependent pathway

**Figure 3:**
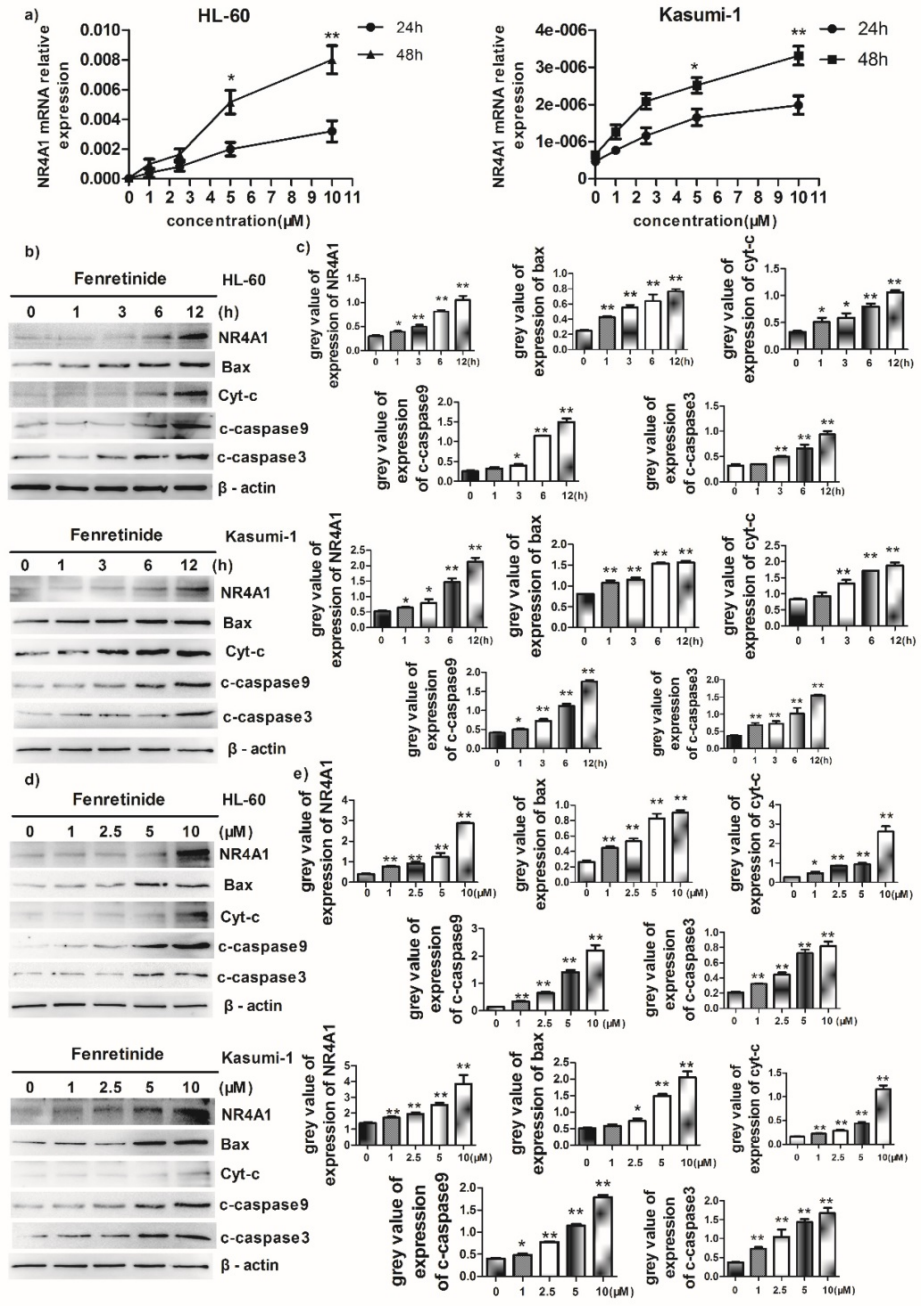

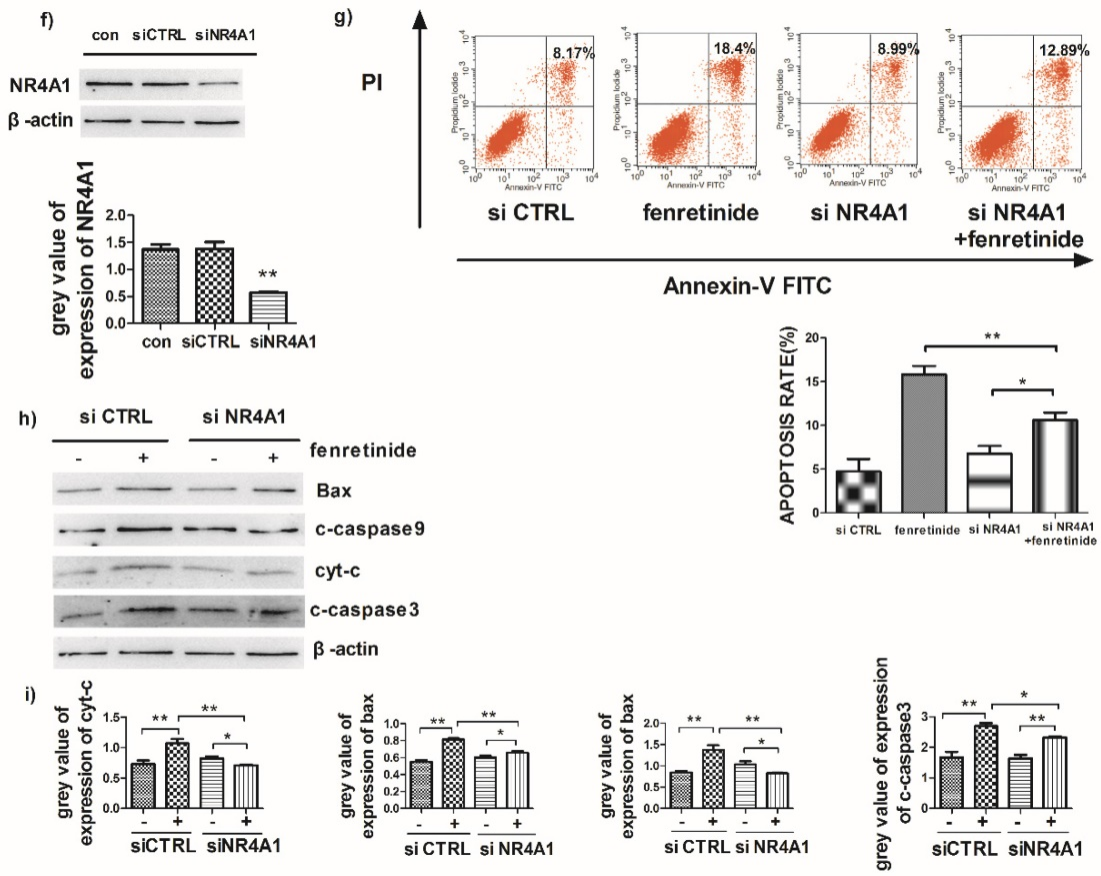
** Fenretinide-induced apoptosis is mediated by NR4A1 and the mitochondria-dependent pathway. a)** NR4A1 mRNA expression is measured using qRT-PCR. HL-60 and Kasumi-1 cells are incubated with fenretinide for different time periods and at various concentrations. Cells are harvested to extract RNA, followed by subsequent reverse transcription into cDNA. **b)** HL-60 and Kasumi-1 cells are treated with 10 μM fenretinide for different time periods. Then, cell lysates re subjected to Western blotting to analyze NR4A1 and the mitochondria-mediated pathway-related proteins. All data represent the means of 3 independent experiments. **c)** Grey value of protein expression in b). **d)** HL-60 and Kasumi-1 cells are treated with fenretinide at various concentrations, and the processing method is the same as b). All data are representative of 3 independent experiments. **e)** Grey value of protein expression in d). **f)** The NR4A1 level is measured using Western blotting after HL-60 cells are transfected with siCTRL or siNR4A1 for 24 h. The blots represent the means of three independent experiments. **g)** HL-60 cells are transfected with siCTRL or siNR4A1 for 12 h and then treated with 10 μM fenretinide for 12 h. siCTRL is used as the negative control. Apoptotic cells are tested using flow cytometry. Data are presented as means ± SD. *p≤0.05; **p≤0.01. **h)** HL-60 cells are transfected with siCTRL or siNR4A1 for 12h and then treated with 10 μM fenretinide for 12 h. Later, cell lysates are collected for Western blotting to determine the expression of apoptosis-related proteins. **i)** Grey value of protein expression in h). All data are expressed as mean ± SEM of tests carried out in triplicate.

Abnormal NR4A1 expression has been detected in multiple human cancer types. Besides, the abrogation or reduction in NR4A gene dosage promotes the development of hematopoietic neoplasms in mice^13^,^14^. Also, other studies show that NR4A1 plays an important role in the chemotherapeutics-induced apoptosis. Therefore, the effect of fenretinide on NR4A1 expression at mRNA and protein levels was explored in this study. The NR4A1 mRNA expression was up-regulated in a time- and concentration-dependent manner in both HL-60 and Kasumi-1 cells after fenretinide treatment (Figure 3a). Typically, NR4A1 induction by fenretinide began at 1 h, which peaked at 12 h (Figure 2b and 3c); afterwards, NR4A1 expression began to decrease (data not shown), and NR4A1 expression was also increased in a concentration-dependent manner (1 to 10 μM) (Fig. 2d and 2e). One mitochondria-mediated pathway is reported to be involved in the NR4A1-induced apoptosis of cancer cells^29^. In our research, the expression levels of relevant proteins involved in the mitochondria-mediated pathway were detected in HL-60 and Kasumi-1 cells after fenretinide treatment (Fig. 2b and 2d), and it was shown that this effect was similar to that of NR4A1.

To further explore the role of NR4A1 in the fenretinide-induced apoptosis of AML cell lines, siNR4A1 was adopted to knock down NR4A1 (Fig. 3f). Flow cytometry analysis confirmed that the apoptosis rate was declined after siNR4A1 transfection into HL-60 and Kasumi-1 cells treated by fenretinide (Fig. 3g). Accordingly, the expression of mitochondria-mediated pathway-associated proteins was decreased after siNR4A1 transfection (Fig. 3h).

### Fenretinide induced NR4A1 translocation into mitochondria and Bcl-2 transformation

**Figure 4:**
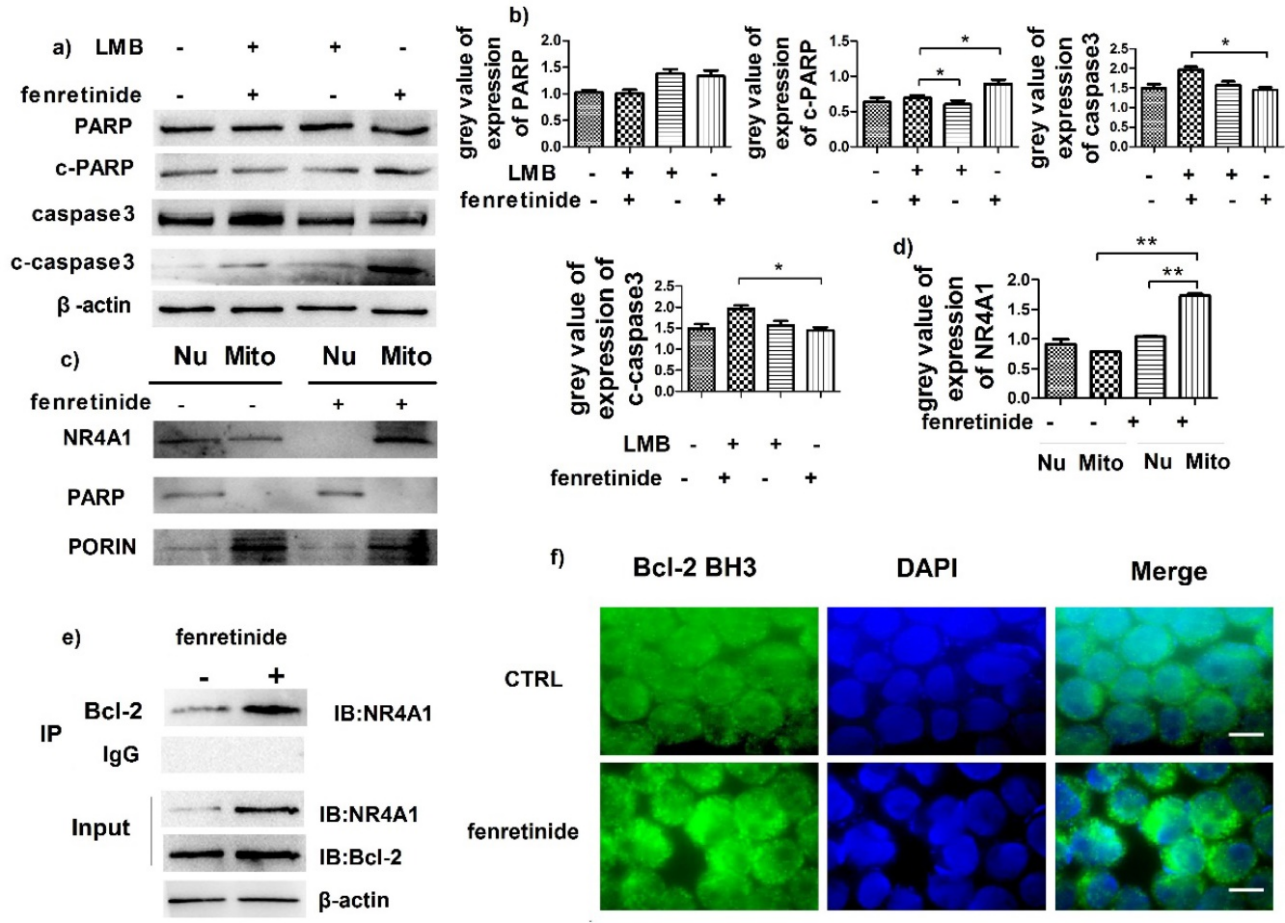
** Fenretinide induces the translocation of NR4A1 into mitochondria and the transformation of Bcl-2. a)** HL-60 cells are treated with 10 μM fenretinide in the presence or absence of LMB for 48 h. Cell lysates are then collected for Western blotting, so as to examine the expression of the cleaved PARP and caspase-3. β-actin is used as a loading control. **b)** Grey value of protein expression in a). **c)** HL-60 cells are treated with or without 10 μM fenretinide for 12 h, and then the mitochondria and nuclei are extracted to test the NR4A1 expression levels. PARP and porin are used as the loading controls. **d)** Grey value of NR4A1 expression in c). **e)** HL-60 cells are treated with 10 μM fenretinide for 12 h. Cell lysates are immunoprecipitated with an anti-Bcl-2 antibody (IP Bcl-2), and NR4A1 is detected using Western blotting. Inputs of cell lysates without IP process are used as the positive controls. **f)** HL-60 cells are treated with 10 μM fenretinide for 12 h. Immunofluorescence staining of Bcl-2 BH3 is performed as described in Materials and methods. The images shown are under 1000× magnification. Scale bars, 20 μm. The images are representative of three independent experiments.

NR4A1 exerts a pro-apoptotic effect through nuclear export. To determine the influence of such effect, HL-60 cells were pretreated with leptomycin B (LMB, an inhibitor of nuclear export) prior to incubation with fenretinide. Our results suggested that fenretinide exposure augmented the expression of c-PARP and c-caspase3 in HL-60 cells, which was down-regulated upon LMB pretreatment (Fig. 4a and 4b), suggesting that the fenretinide-induced apoptosis was dependent on nuclear export.

Some evidence suggests that the subcellular translocation of NR4A1 from nuclei into mitochondria determines its role in cell survival and death. Hence, the effect of NR4A1 translocation after fenretinide treatment on HL-60 cells was determined through Western blotting of the subcellular fractions isolated from HL-60 cells with or without fenretinide treatment. Typically, Porin and PARP were used as the markers of nuclei and mitochondria, respectively. As shown in Fig. 4c and 4d, NR4A1 is distributed in both nuclei and mitochondria before fenretinide treatment. By contrast, NR4A1 expression was primarily found in mitochondria after fenretinide treatment in HL-60 cells, suggesting that fenretinide induced NR4A1 to translocate from nuclei into mitochondria.

NR4A1 can interact with Bcl-2 in mitochondria, which will give rise to the conformational changes in Bcl-2 to expose its BH3 domain, which in turn changes the function of Bcl-2 from anti-apoptotic to pro-apoptotic^30^,^31^. To find out whether NR4A1 interacted with Bcl-2 in AML cells after fenretinide treatment, co-immunoprecipitation experiments were performed to measure the intensity of interaction between NR4A1 and Bcl-2. As displayed in Figure 4e, fenretinide augments the intensity of interaction between NR4A1 and Bcl-2 in HL-60 cells. To further test the effect of fenretinide on the conformational changes of Bcl-2, immunofluorescence staining was carried out to determine the exposure of Bcl-2 BH3 domain using the Bcl-2 (BH3) antibody. According to Figure 4f, the intensity of Bcl-2 BH3 immunofluorescence staining is increased after fenretinide treatment in HL-60 cells. These results suggested that fenretinide promoted the binding of NR4A1 to Bcl-2, which then resulted in the conformational changes in Bcl-2 to expose its BH3 domain.

### Fenretinide + ketoconazole was active against mouse xenografts

**Figure 5:**
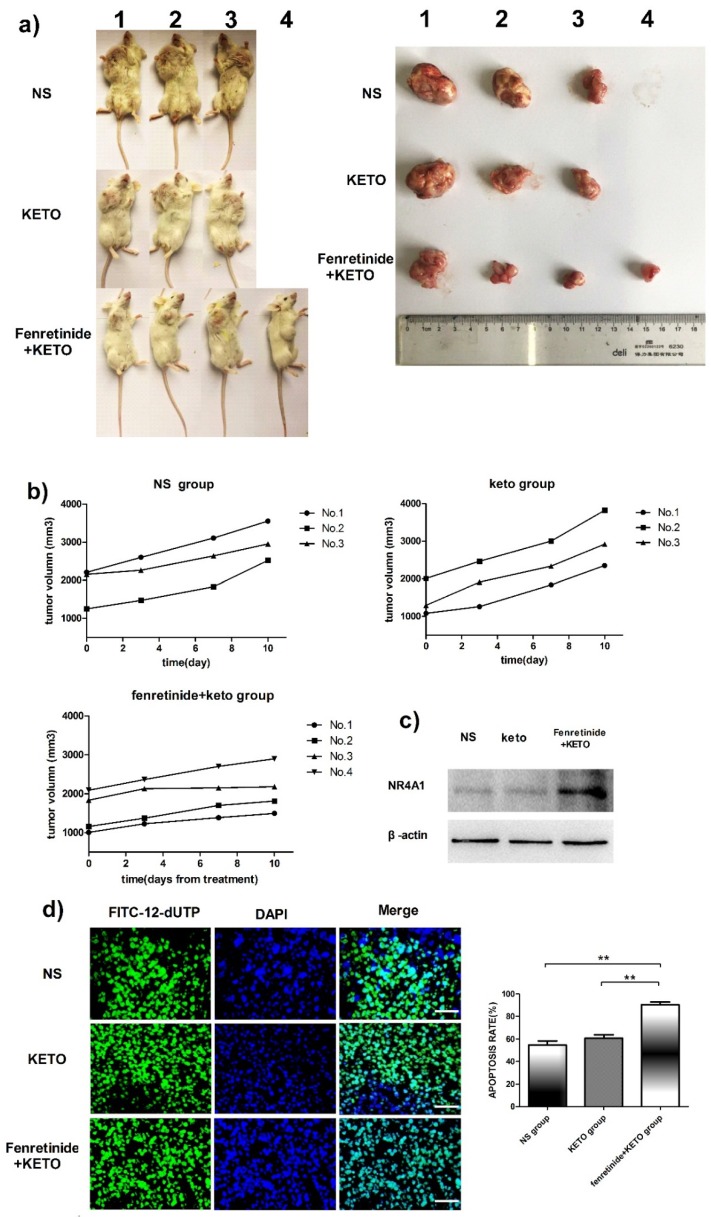
** Fenretinide + ketoconazole is found to be active against mouse xenografts.** NOD/SCID mice that are subcutaneously injected with HL-60 cells are given gavage of fenretinide + KETO, KETO alone or NS for 1 week. **a)** Comparison of tumor images. **b)** Tumor volume is compared among the fenretinide + KETO, KETO alone and NS groups. **c)** NR4A1 expression in tumors is analyzed through Western blotting. The blots are representative of three independent experiments. **d)** TUNEL assay is carried out according to the TUNEL apoptosis assay. The images shown are under 400× magnification. Scale bars, 20 μm.The number of apoptosis-positive cells is counted in five high-power fields (400× magnification), and the mean percentage of apoptotic cells is recorded. **p<0.001.

The activity of fenretinide *in vivo* against the subcutaneous murine xenograft models was also examined. Fenretinide administration via continuous infusion (to achieve a plasma level of 40 mmol/L in patients^26^) is not feasible in mice. As a result, the LXS fenretinide oral powder^27^ was used in this study, which was given in combination with KETO. KETO increased the fenretinide plasma levels in mice from 10 mmol/L to 20 mmol/L, which was achieved through inhibiting fenretinide metabolism^28^. Figure 5a shows the images of tumors in the xenograft models. The tumor volume in fenretinide + KETO group was lower than that in KETO alone or NS group (Fig. 5b). To determine the influence of fenretinide on NR4A1 expression in NOD/SCID mice, Western blotting analysis was performed on tumor tissues collected from the NS, KETO and fenretinide + KETO groups using HL-60 cell injection. NR4A1 expression was up-regulated in fenretinide + KETO group compared with that in the other two groups (Fig. 5c). Moreover, the TUNEL assay was utilized to determine whether administration of fenretinide + KETO inhibited tumor growth through inducing the apoptosis of tumor cells. The fenretinide + KETO group had a remarkably higher apoptosis-positive cell count relative to those in the other two groups (p<0.001; Fig. 5d).

## Discussion

Fenretinide, a synthetic derivative of retinoic acid, promotes growth inhibition and induces apoptosis in numerous tumor cell types. Evidence supports that loss of NR4A1 is highly correlated with the development of AML, while restoration of NR4A1 is recognized as a promising molecular target for AML intervention^11^,^13^,^29^. This study aimed to examine the effects of NR4A1 activation and fenretinide on the apoptotic activity in AML cells via the mitochondria-mediated mechanisms.

First of all, the effect of fenretinide on suppressing the proliferation of AML cells was examined. We found that fenretinide inhibited the viability of both HL-60 and Kasumi-1 cells. Specifically, the approximate IC50 at 48 h was 6.18 μM in HL-60 cells and 6.75 μM in Kasumi-1 cells, suggesting that HL-60 cells had a higher sensitivity to fenretinide than that reported by Morad et al. (IC50 7.5 μM at 72 h)^32^. Our results were consistent with those from Jiang (87.77% viable HL60 cells at 24 h and 36.91% at 48 h)^33^. Subsequently, the apoptotic effect of fenretinide on HL-60 and Kasumi-1 cells was also examined. Flow cytometry analysis revealed that the apoptosis rates of HL-60 and Kasumi-1 cells treated with fenretinide were in line with the results from cell viability experiments, revealing that apoptosis was the main type of cell death induced by fenretinide.

Afterwards, the effect of fenretinide on NR4A1 expression in HL-60 and Kasumi-1 cells was also determined. Western blotting showed that fenretinide immediately and transiently induced NR4A1 expression in a time- and concentration-dependent manner, which acted as an immediate early gene. Fenretinide substantially induced the expression of cyt-c, Bax, c-caspase-9 and c-caspase-3. In addition, siNR4A1 not only decreased the fenretinide-induced apoptosis, but also reversed the expression of mitochondria-mediated apoptosis-related proteins, demonstrating that the mitochondria-mediated pathway was involved in the fenretinide-induced apoptosis of AML cells. The murine subcutaneous AML xenograft models were also employed to assess the activity of fenretinide *in vivo*. The results indicated that fenretinide at an effective blood concentration induced NR4A1 expression, reduce tumor burden, and promote the apoptosis of AML cells in AML mouse models.

A growing body of evidence has demonstrated that cyt-c release and caspase precursors are activated in response to certain stimuli through NR4A1 translocation from nuclei to mitochondria, where NR4A1 binds to the N-terminal of Bcl-2, thus exposing the BH3 domain of Bcl-2. Such conformational changes in Bcl-2 will in turn bring about the conversion of Bcl-2 from an anti-apoptotic to a pro-apoptotic state, thereby triggering cyt-c activation and the apoptosis cascade^30^,^31^. Therefore, we wondered whether NR4A1 nuclear export and the subsequent Bcl-2 transformation were involved in the fenretinide-induced apoptosis of AML cells. According to our results, fenretinide activated the expression of c-PARP and c-caspase3, which was blocked by LMB, an inhibitor of nuclear export. Furthermore, fenretinide promoted NR4A1 expression in mitochondria and reduced that in the nuclei of HL-60 cells, suggesting that fenretinide-induced apoptosis might be ascribed to NR4A1 translocation from nuclei into mitochondria. Additionally, results of co-immunoprecipitation and immunofluorescence revealed that fenretinide promoted the interaction between NR4A1 and Bcl-2, which exposed the BH3 domain of HL-60 cells, indicating that the NR4A1-mediated conformational changes in Bcl-2 might be involved in the fenretinide-caused apoptosis effect on AML cells. It is generally agreed that, fenretinide is a reactive oxygen species (ROS) generator, and that ROS production plays a central role in the fenretinide-induced apoptotic process. However, findings in this study demonstrated that the NR4A1-mediated mitochondrial pathway played a major role in the fenretinide-induced apoptosis of AML cells. It has been reported that, the NR4A1 export-related apoptosis is regulated by its heterodimerization with retinoid X receptor alpha (RXRα) in numerous cancer cells^34^. Fenretinide transactivates the RXRα/RARβ-mediated pathway and directly increases the transcriptional activity of RARβ (retinoic acid receptor β). Besides, the knockdown of RARβ mRNA expression evidently impairs the fenretinide-induced apoptosis in Huh-7 cells^35^, while fenretinide-induced RARβ either directly or indirectly interacts with NR4A1. Such interaction can stabilize the NR4A1 protein levels, and the RARβ/NR4A1 protein complex is then exported to the cytosol, which most likely targets the mitochondria to induce apoptosis^36^.

To sum up, our data suggest that NR4A1 plays a crucial role in the fenretinide-induced apoptosis of AML cells. Therefore, targeting NR4A1 may be a potential intervention to treat AML.

## Figures and Tables

**Table 1:** The characteristics of patients with AML and healthy donors

Patients		Age (year)	gender	FAB	WBC (×10^9^ /L)	HB (g/L)	PLT (×10^9^ /L)	%Blasts (Bone Marrow)	Cytogene-tics	Mutations /fusion gene
1		16	male	M2	17	44	4	71	normal	normal
2		30	female	M2	5.78	64	27	54	normal	normal
3		43	female	M2	134	88	80	86	8 trisome	normal
4		30	female	M2	30	79	15	76	normal	normal
5		53	female	M2	53.2	84	211	87	normal	normal
6		19	male	M2	218	92	124	76.5	normal	FLT3-ITD
7		85	male	M2	1.13	74	47	30	normal	normal
8		37	female	M2	8.88	138	199	21	t(8,21)	AML1-ETO
9		22	male	M3	21	79	13	57	t(15,17)	PML-RARα
10		37	male	M3	28.24	60	43	94	t(15,17)	PML-RARα
11		27	male	M3	1.0	139	68	83	t(15,17)	PML-RARα
12		55	female	M3	58.35	62	24	27	t(15,17)	PML-RARα
13		66	female	M4	22.5	67	16	69	t(8,21)	AML1-ETO
14		77	female	M4	75.81	66	47	82	normal	normal
15		17	female	M4	177	77	13	57	normal	normal
16		49	male	M4	65.37	75	29	61	normal	normal
17		34	male	M4	55.4	57	77	78	normal	normal
18		78	male	M5	43.5	98	100	68	normal	normal
19		39	male	M5	79.2	41	56	73	normal	normal
20		54	male	M5	135.32	70	40	73	normal	normal
21		76	female	M5	25.89	58	4	42	normal	normal
Donors										
1		46	female	-	6.86	112	216	-	-	-
2		44	male	-	5.25	154	189	-	-	-
3		28	male	-	5.49	172	381	-	-	-
4		25	female	-	7.03	137	229	-	-	-
5		22	female	-	6.95	137	178	-	-	-
6		24	male	-	6.98	169	255	-	-	-

## Abbreviations

AML: Acute myeloid leukemia
Allo-HSCT: allogeneic hematopoietic stem cell transplantation
ATRA: all-trans retinoic acid
CCK-8: cell-counting kit-8
CR1: first complete remission
cyt-c: cytochrome C
HSCT: allogeneic hematopoietic stem cell transplantation
IC50: half maximal inhibitory concentration
IP: immunoprecipitation
KETO: ketoconazole
MDS/MPN: myelodysplastic/ myeloproliferative neoplasms
LMB: leptomycin B
MNCs: mononuclear cells
RARβ: retinoic acid receptor β
ROS: reactive oxygen species
RXRα: retinoid X receptor alpha

## References

[1] Walter RB, Estey EH (2015). Management of older or unfit patients with acute myeloid leukemia. *Leukemia*.

[2] Siegel RL, Miller KD, Jemal A (2018). Cancer statistics, 2018. *CA Cancer J Clin*.

[3] Estey EH (2013). Acute myeloid leukemia: 2013 update on risk-stratification and management. *Am J Hematol*.

[4] Alibhai SM, Leach M, Minden MD, Brandwein J (2009). Outcomes and quality of care in acute myeloid leukemia over 40 years. *Cancer*.

[5] Juliusson G, Antunovic P, Derolf A (2009). Age and acute myeloid leukemia: real world data on decision to treat and outcomes from the Swedish Acute Leukemia Registry. *Blood*.

[6] Meyers J, Yu Y, Kaye JA, Davis KL (2013). Medicare fee-for-service enrollees with primary acute myeloid leukemia: an analysis of treatment patterns, survival, and healthcare resource utilization and costs. *Appl Health Econ Health Policy*.

[7] Oran B, Weisdorf DJ (2012). Survival for older patients with acute myeloid leukemia: a population-based study. *Haematologica*.

[8] Estey E (2016). Acute myeloid leukemia: 2016 Update on risk-stratification and management. *Am J Hematol*.

[9] Pawlak A, Strzadala L, Kalas W (2015). Non-genomic effects of the NR4A1/Nur77/TR3/NGFIB orphan nuclear receptor. *Steroids*.

[10] Wang WJ, Wang Y, Chen HZ (2014). Orphan nuclear receptor TR3 acts in autophagic cell death via mitochondrial signaling pathway. *Nat Chem Biol*.

[11] Wenzl K, Troppan K, Neumeister P, Deutsch AJ (2015). The nuclear orphan receptor NR4A1 and NR4A3 as tumor suppressors in hematologic neoplasms. *Curr Drug Targets*.

[12] Lee SO, Li X, Khan S, Safe S (2011). Targeting NR4A1 (TR3) in cancer cells and tumors. *Expert Opin Ther Targets*.

[13] Mullican SE, Zhang S, Konopleva M (2007). Abrogation of nuclear receptors Nr4a3 and Nr4a1 leads to development of acute myeloid leukemia. *Nat Med*.

[14] Ramirez-Herrick AM, Mullican SE, Sheehan AM, Conneely OM (2011). Reduced NR4A gene dosage leads to mixed myelodysplastic/myeloproliferative neoplasms in mice. *Blood*.

[15] Yang H, Bushue N, Bu P, Wan YJ (2010). Induction and intracellular localization of Nur77 dictate fenretinide-induced apoptosis of human liver cancer cells. *Biochem Pharmacol*.

[16] Schneider BJ, Worden FP, Gadgeel SM (2009). Phase II trial of fenretinide (NSC 374551) in patients with recurrent small cell lung cancer. *Invest New Drugs*.

[17] Zou C, Guan Y, Zou C (2002). N-(4-hydroxyphenyl)retinamide (4-HPR) modulates GADD45 expression in radiosensitive bladder cancer cell lines. *Cancer Lett*.

[18] Hail N Jr, Chen P, Kepa JJ, Bushman LR, Shearn C (2010). Dihydroorotate dehydrogenase is required for N-(4-hydroxyphenyl)retinamide-induced reactive oxygen species production and apoptosis. *Free Radic Biol Med*.

[19] Benelli R, Monteghirfo S, Vene R, Tosetti F, Ferrari N (2010). The chemopreventive retinoid 4HPR impairs prostate cancer cell migration and invasion by interfering with FAK/AKT/GSK3beta pathway and beta-catenin stability. *Mol Cancer*.

[20] Gopal AK, Pagel JM, Hedin N, Press OW (2004). Fenretinide enhances rituximab-induced cytotoxicity against B-cell lymphoma xenografts through a caspase-dependent mechanism. *Blood*.

[21] Abou-Issa H, Moeschberger M, el-Masry W, Tejwani S, Curley RW Jr, Webb TE (1995). Relative efficacy of glucarate on the initiation and promotion phases of rat mammary carcinogenesis. *Anticancer Res*.

[22] McCormick DL, Bagg BJ, Hultin TA (1987). Comparative activity of dietary or topical exposure to three retinoids in the promotion of skin tumor induction in mice. *Cancer Res*.

[23] Ohshima M, Ward JM, Wenk ML (1985). Preventive and enhancing effects of retinoids on the development of naturally occurring tumors of skin, prostate gland, and endocrine pancreas in aged male ACI/segHapBR rats. *J Natl Cancer Inst*.

[24] Garaventa A, Luksch R, Lo Piccolo MS (2003). Phase I trial and pharmacokinetics of fenretinide in children with neuroblastoma. *Clin Cancer Res*.

[25] Puntoni M, Petrera M, Campora S (2016). Prognostic Significance of VEGF after Twenty-Year Follow-up in a Randomized Trial of Fenretinide in Non-Muscle-Invasive Bladder Cancer. *Cancer Prev Res (Phila)*.

[26] Mohrbacher AM, Yang AS, Groshen S (2017). Phase I Study of Fenretinide Delivered Intravenously in Patients with Relapsed or Refractory Hematologic Malignancies: A California Cancer Consortium Trial. *Clin Cancer Res*.

[27] Colombo N, Formelli F, Cantu MG (2006). A phase I-II preoperative biomarker trial of fenretinide in ascitic ovarian cancer. *Cancer Epidemiol Biomarkers Prev*.

[28] Tomayko MM, Reynolds CP (1989). Determination of subcutaneous tumor size in athymic (nude) mice. *Cancer Chemother Pharmacol*.

[29] Li H, Kolluri SK, Gu J (2000). Cytochrome c release and apoptosis induced by mitochondrial targeting of nuclear orphan receptor TR3. *Science*.

[30] Lin B, Kolluri SK, Lin F (2004). Conversion of Bcl-2 from protector to killer by interaction with nuclear orphan receptor Nur77/TR3. *Cell*.

[31] Kolluri SK, Zhu X, Zhou X (2008). A short Nur77-derived peptide converts Bcl-2 from a protector to a killer. *Cancer Cell*.

[32] Morad SA, Davis TS, Kester M, Loughran TP Jr, Cabot MC (2015). Dynamics of ceramide generation and metabolism in response to fenretinide-Diversity within and among leukemia. *Leuk Res*.

[33] Jiang L, Pan X, Chen Y, Wang K, Du Y, Zhang J (2011). Preferential involvement of both ROS and ceramide in fenretinide-induced apoptosis of HL60 rather than NB4 and U937 cells. *Biochem Biophys Res Commun*.

[34] Maurer BJ, Kalous O, Yesair DW (2007). Improved oral delivery of N-(4-hydroxyphenyl)retinamide with a novel LYM-X-SORB organized lipid complex. *Clin Cancer Res*.

[35] Cooper JP, Hwang K, Singh H (2011). Fenretinide metabolism in humans and mice: utilizing pharmacological modulation of its metabolic pathway to increase systemic exposure. *Br J Pharmacol*.

[36] Boudreaux SP, Ramirez-Herrick AM, Duren RP, Conneely OM (2012). Genome-wide profiling reveals transcriptional repression of MYC as a core component of NR4A tumor suppression in acute myeloid leukemia. *Oncogenesis*.

